# Women and men with distressing low sexual desire exhibit sexually dimorphic brain processing

**DOI:** 10.1038/s41598-024-61190-4

**Published:** 2024-05-14

**Authors:** Natalie Ertl, Edouard G. Mills, Matthew B. Wall, Layla Thurston, Lisa Yang, Sofiya Suladze, Tia Hunjan, Maria Phylactou, Bijal Patel, Paul A. Bassett, Jonathan Howard, Eugenii A. Rabiner, Ali Abbara, David Goldmeier, Alexander N. Comninos, Waljit S. Dhillo

**Affiliations:** 1 Invicro London, London, UK; 2 Section of Endocrinology and Investigative Medicine, Imperial College London, London, UK; 3 Statsconsultancy Ltd, Amersham, UK; 4 Jane Wadsworth Sexual Function Clinic, Imperial College Healthcare NHS Trust, London, UK; 5 Department of Endocrinology, Imperial College Healthcare NHS Trust, London, UK

**Keywords:** Sexual behaviour, Sexual dimorphism

## Abstract

Distressing low sexual desire, termed Hypoactive Sexual Desire Disorder (HSDD), affects approximately 10% of women and 8% of men. In women, the ‘top-down’ theory of HSDD describes hyperactivity in higher-level cognitive brain regions, suppressing lower-level emotional/sexual brain areas. However, it is unknown how this neurofunctional disturbance compares to HSDD in men. To investigate this, we employed task-based functional MRI in 32 women and 32 men with HSDD to measure sexual-brain processing during sexual versus non-sexual videos, as well as psychometric questionnaires to assess sexual desire/arousal. We demonstrate that women had greater activation in higher-level and lower-level brain regions, compared to men. Indeed, women who had greater hypothalamic activation in response to sexual videos, reported higher psychometric scores in the evaluative (r = 0.55, P = 0.001), motivational (r = 0.56, P = 0.003), and physiological (r = 0.57, P = 0.0006) domains of sexual desire and arousal after watching the sexual videos in the scanner. By contrast, no similar correlations were observed in men. Taken together, this is the first direct comparison of the neural correlates of distressing low sexual desire between women and men. The data supports the ‘top-down’ theory of HSDD in women, whereas in men HSDD appears to be associated with different neurofunctional processes.

## Introduction

Sexual desire plays a pivotal role in the human experience, contributing to emotional bonding, intimacy, and overall well-being^1,2^. Hypoactive Sexual Desire Disorder (HSDD) is among the most common sexual health complaints, and is characterized by a persistent lack of sexual interest or desire causing marked distress to the individual^3,4^. HSDD affects approximately 10% of women^5^ and 8% of men^6^. While the disorder as a whole is relatively under-studied, the extant work has been overwhelmingly dedicated to understanding HSDD in women. The current neurofunctional theory of HSDD is the 'top-down' theory, relating to excessive hyperactivation of higher-level cortical brain regions (regulating introspection, self-monitoring, and feelings of guilt), which leads to a hypoactivation of lower-level limbic regions (governing emotion, reward, and sexual processing)^7^. However, the top-down theory is based *exclusively* on research in women with HSDD. By contrast, limited work has examined the neural mechanisms which may be involved in HSDD in men. One positron emission tomography study (as opposed to the functional MRI (fMRI) modality used in studies in women) demonstrated that men with HSDD do *not* deactivate their medial orbitofrontal cortex in response to sexual images, whereas healthy men do^8^. This disparity in research is reflected in the current treatment options available for HSDD. There are two licenced options available for women with HSDD in the United States, but crucially none for men. Furthermore, in men, HSDD is frequently misdiagnosed as erectile dysfunction with this misdiagnosis and subsequent mistreatment often intensifying the distress^9^.

To this end, directly comparing the neurobiological basis of HSDD in both sexes is essential for furthering our understanding of the disorder which could lead to targeted and effective treatments and interventions. By examining functional brain responses in both women and men with HSDD for the first time, this study aims to advance our understanding of the neural mechanisms that govern sexual responses in these patients, with the ultimate goal of informing sex-specific therapeutic approaches. To examine this, we employed fMRI during a visual sexual stimuli task designed to trigger underlying sexual-brain processing in 32 women with HSDD and 32 men with HSDD. The task consisted of sexual and control (exercise) videos. To provide functional and behavioural relevance for the brain activity changes, participants also completed well-established psychometric questionnaires.

## Results

### Demographics

Baseline demographics for the participants can be found in Table 1. The women were younger than the men, however, the participants did not differ significantly in the number of times they engaged in intercourse per month, duration of relationship, or depression and anxiety measures.

**Table 1:** Participant baseline characteristics. PHQ-9 = Patient Health (Depression) Questionnaire-9, max score 27; GAD7 = Generalised Anxiety Disorder-7 assessment, max score 21, N = 32 women and N = 32 men.

	Women	Men	
Age (years) (mean, SD)	29.16 6.73	37.91 8.61	t[62] = 4.53, P < 0.001***
BMI (kg/m^2^) (mean, SD)	23.14 2.73	24.93 5.40	t[62] = 1.67, P = 0.100
Sexual intercourse (per month) (mean, SD)	2.51 1.47	2.53 2.67	t[62] = 0.04, P = 0.965
Duration of relationship (years) (mean, SD)	5.13 3.53	7.78 7.54	t[62] = 1.80, P = 0.077
PHQ-9 (mean, SD)	2.28 2.46	2.47 2.46	t[62] = 0.31, P = 0.756
GAD7 (mean, SD)	2.59 2.31	1.94 2.56	t[62] = -1.08, P = 0.286

### Whole-brain analysis comparison

**Figure 1:**
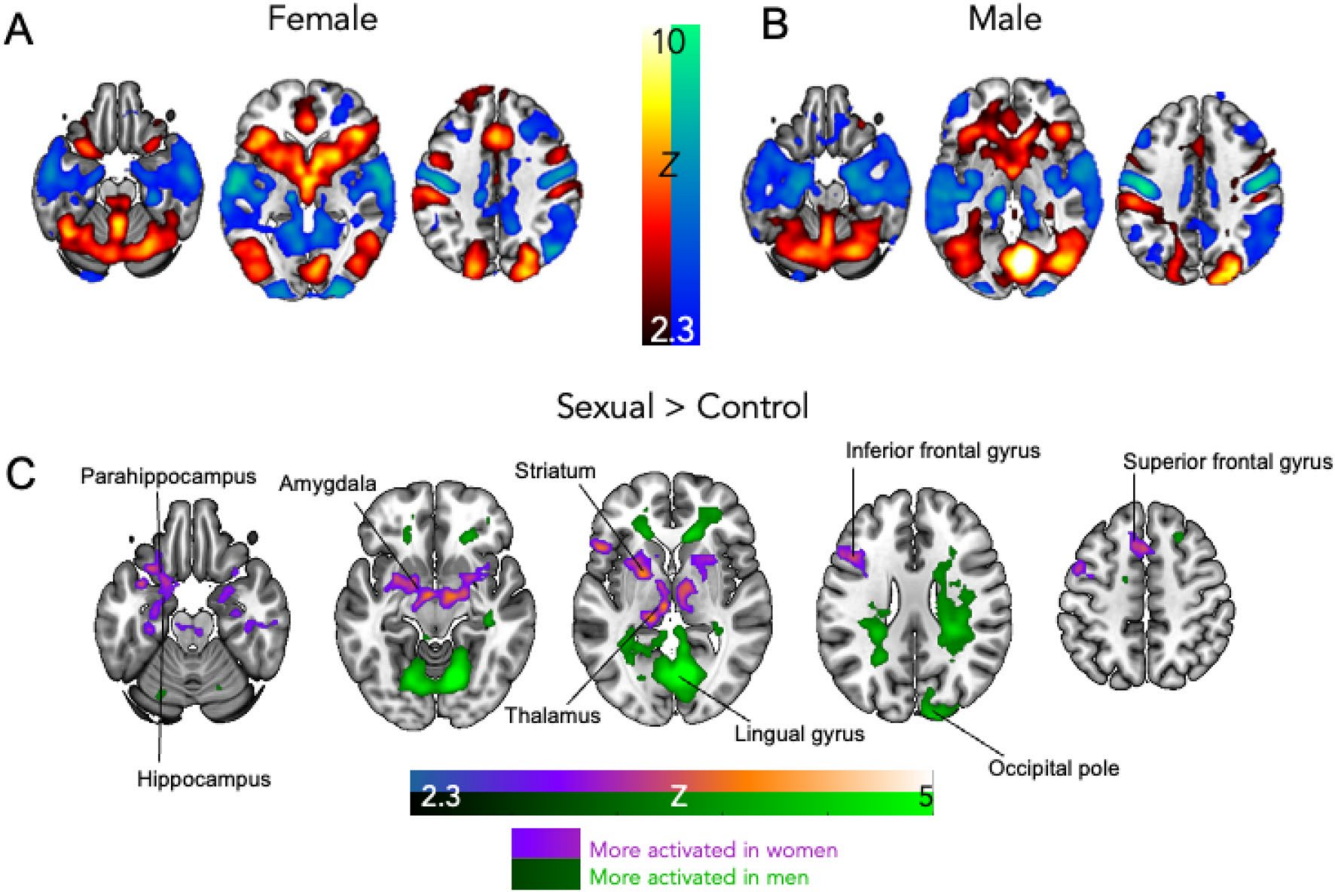
Women with HSDD have greater limbic activation to sexual videos than men. (**A**) The female and (**B**) male HSDD group average results showing the brains activation (red/yellow) and deactivation (blue green) to sexual compared to control (exercise) videos. (**C**) Brain regions more activated in women (relative to men) to sexual compared to control videos are shown in purple. Brain regions more activated in men (relative to women) to sexual compared to control videos are shown in green. Results are cluster corrected and thresholded to Z = 2.3, P < 0.05, N = 64 (32 women, 32 men).

The overall effects of the sexual-brain processing task were similar in both the women and men with HSDD (Fig. 1A,B). When comparing the sexual videos compared to control videos, activation (shown in red/yellow) occurred in the striatum, visual cortex, cerebellum, and anterior cingulate (areas which are well-established to be involved in sexual-brain processing^10,11^). In addition, when comparing sexual videos to control videos, there were areas where there was less activation when watching sexual compared to control videos, defined as deactivation (shown in blue/green). Deactivation was observed around the temporal cortex, posterior cingulate, and motor cortex (Fig. 1A,B).

In order to directly compare the similarities in the spatial pattern of neural responses, between the women and men to sexual compared to control videos, the data were binarised to produce masks of the task effects (task effects shown in Fig. 1A,B and for additional brain slices of task effects see Supplementary Fig. 1), meaning voxels where there is (de)activation were set to a value of 1 and all other voxels retained a value of 0. These binarised images from the women and men’s data were then multiplied together. Since 1 × 1 = 1 and 1 × 0 = 0, voxels where there is activation in both women *and* men will retain a value of 1 (with all other voxels being 0) and so an overlap image of spatial (de)activation was produced (Supplementary Fig. 1). The overlap image shows the common areas in the spatial pattern of activation to sexual and control videos in both women *and* men. Dice coefficients were then calculated to quantify the overlap in each contrast: activation (sexual > control) = 0.601, deactivation (control > sexual) = 0.643. As stated by Wüthrich et al.^12^, there is a lack of consensus regarding the interpretation of Dice coefficients. However, as proposed by Wüthrich^12^, we followed the guidelines set out by Cicchetti^13^. Coefficients below 0.40 are considered poor; between 0.40 and 0.59, fair; between 0.60 and 0.74, good, and > 0.75, excellent. Therefore, our results demonstrate good replicability of task effect results and a similar general spatial pattern of responses in both sexes.

After comparing the spatial similarities in neural responses to sexual and control videos, differences between the women and men were then directly compared in their responses to the sexual compared to control videos, and important dimorphisms were observed. Women had significantly greater activation in limbic brain regions (such as the amygdala, striatum, and thalamus), compared to men (purple, Fig. 1C). Furthermore, women also had greater activation in higher-level cortical regions such as the inferior frontal gyrus and the superior frontal gyrus. In contrast, men had greater activation in areas within the visual cortex, such as the lingual gyrus and occipital pole (green, Fig. 1C). A summary table of cluster coordinates can be found in Supplementary Table 1.

### Comparing sexual Regions Of Interest

**Figure 2:**
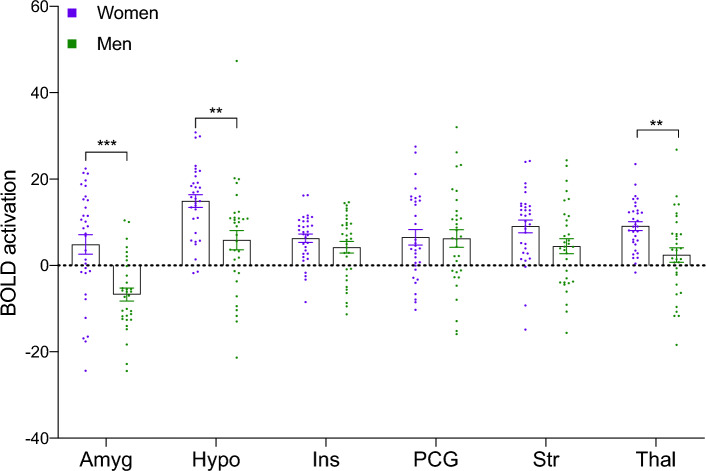
Women with HSDD have greater activation in regions associated with the sex-network than men. Women (purple) had significantly more activation in the amygdala, hypothalamus, and thalamus to sexual (compared to control exercise videos) than men (green). Amyg = Amygdala, Hypo = Hypothalamus, Ins = Insula, PCG = Pre-Central Gyrus, Str = Striatum, Thal = Thalamus. P values corrected for multiple comparisons: P < 0.01*, P < 0.005, ** P < 0.0005***. Error bars show SEM, N = 64 (32 women, 32 men).

To investigate the differences between women and men with HSDD in more detail, standard Regions of Interest (ROIs) from the *sex network* (amygdala, hypothalamus, insula, pre-central gyrus, striatum, and thalamus) were extracted to directly assess for differences between the female and male brain responses to sexual videos. A significant interaction effect (sex (female/male) * ROI) was confirmed using an ANOVA (F[5,102] = 3.87, P = 0.002). Follow-up unpaired t-tests were then performed to identify ROIs driving this effect with a reduced alpha value calculated using the Benjamini–Hochberg False Discovery Rate (FDR) correction to account for multiple comparisons (FDR thresholds of 0.05 *, 0.01 **, and 0.001***). Significant differences were found between women and men in the amygdala (*P_FDR_ < 0.001; t[62] = 4.26, P = 0.00007, adjusted threshold α < 0.00017), hypothalamus (**P_FDR_ < 0.01; t[62] = 3.35, P = 0.0013, adjusted threshold α < 0.0050), and thalamus (**P_FDR_ < 0.01; t[62] = 3.41, P = 0.0011, adjusted threshold α < 0.0033), and a trend in the striatum (P_FDR_ < 0.05; t[62] = 2.02, P = 0.0472, adjusted threshold α < 0.033), with women showing greater activation than men (Fig. 2). To confirm the observed differences were specifically in response to sexual videos and not reflective of general baseline brain activation differences between women and men, the data were extracted and compared from the control video > baseline, as well as the sexual video > baseline contrasts. No significant interaction was identified in the control > baseline comparison (F[5,310] = 1.33, P = 0.249; Supplementary Fig. 2A). However, a significant interaction was identified when viewing the sexual videos > baseline (F[5,310] = 6.02, P < 0.0001) in keeping with the sexual video > control findings above, thereby demonstrating a specificity for this effect to the sexual response. Follow-up unpaired t-tests (with the reduced alpha value calculated using Benjamini–Hochberg FDR) showed the women had significantly more activation in the amygdala (P_FDR_ < 0.001 ***, t[62] = 5.13, P < 0.001, adjusted threshold α < 0.00017), hypothalamus (P_FDR_ < 0.05 *, t[62] = 2.68, P = 0.009, α < 0.025) and striatum (P_FDR_ < 0.05 *, t[62] = 2.71, P = 0.008, α < 0.017), and the thalamus (P_FDR_ < 0.05 *, t[62] = 2.53, P = 0.014, α < 0.033; Supplementary Fig. 2B).

### Correlational analysis

**Figure 3:**
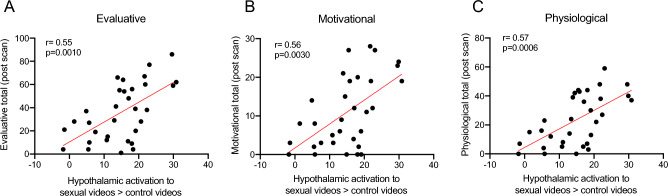
Women with greater activation in sex-specific regions reported greater positive feelings about sex. Activation in response to sexual compared to exercise videos in the hypothalamus correlated with (**A**) total evaluative score, (**B**) total motivational score, (**C**) and total physiological score in the post scan scores of women with HSDD; N = 32.

To provide behavioural relevance for the brain activity data, post-hoc correlations were performed separately in women and men. The post-scan Sexual Arousal and Desire Inventory (SADI) domain psychometric ratings were correlated with the degree of activation in sex-network regions (in response to sexual > control videos). Significant positive correlations were observed between hypothalamic activation and the evaluative (P_FDR_ < 0.05 *, r = 0.55, P = 0.001, adjusted threshold α < 0.0042), motivational (P_FDR_ < 0.05 *, r = 0.56, P = 0.003, adjusted threshold α < 0.0063), and physiological (P_FDR_ < 0.05 *, r = 0.57, P = 0.0006, adjusted threshold α < 0.0021) SADI domains in the women, (Fig. 3).

No significant correlations were found between the men’s SADI domain scores and the degree of activation in any of the sexual-brain ROIs.

## Discussion

This study presents the first direct comparison of the neural correlates of distressing low sexual desire between women and men, identifying sexual dimorphisms in the neurobiological network underlying the abnormal sexual responses in HSDD. The findings are somewhat consistent with previous research in individuals with normal sexual desire, suggesting that women and men exhibit similar general overall patterns of activation to visual sexual stimuli^11,14^. However, notable differences were observed in the activation of limbic brain regions in women and men with HSDD, particularly the hypothalamus, amygdala, and thalamus, which are key structures associated with emotional processing and sexual motivation^15,16^. Indeed, previous fMRI studies have identified the hypothalamus^14,17,18^, amygdala^14^, and thalamus^17^ as regions more strongly activated in response to sexual stimuli in healthy men compared to healthy women. By contrast, here we found that women with HSDD demonstrate a greater magnitude of activation in these three regions than the men with HSDD. Furthermore, we confirmed that these findings were specifically in response to visual sexual stimuli as no differences were identified in the control comparison (exercise > baseline). Therefore, given that these brain regions are known to play a pivotal role in the motivational and emotional components of sexual arousal^19^, this provides possible evidence for their involvement in the neural bases underlying HSDD in men. Furthermore, previous work has identified women with HSDD as having lower activation in limbic regions in response to sexual stimuli compared to *healthy* women^7^. Our data therefore presents a possible hierarchy of activation in limbic regions to sexual stimuli: healthy men, healthy women, women with HSDD, and finally men with HSDD having the lowest activation in emotional brain regions to sexual stimuli.

This sexual dimorphism highlights the need for more research to be conducted into men with HSDD. The theoretical model of HSDD presented by Cacioppo suggests that women with HSDD have hyperactivation in higher-level cortical regions, which then leads to hypoactivation in limbic regions of the brain in a ‘top-down’ inhibition of the sexual response^7^. The present correlations from our data in women support this hypothesis, since women with lower activation in the hypothalamus reported lower scores on sex-positive scales in the SADI questionnaires. This suggests that women with more hypoactivation in this limbic sex-brain-region feel less positive about sex, and perhaps have more severe HSDD. We also identified significantly greater activation in the left inferior-frontal gyrus in the women with HSDD relative to the men with HSDD. Consistent with this, the left inferior frontal gyrus is an area associated with internal monologuing^20^, feelings of guilt^21^, and has been previously identified as a region which is hyperactive in women with HSDD^7^.

By contrast, men exhibited significantly greater visual activation in response to sexual videos (compared to control exercise videos) than women. This heightened activation in the visual cortex aligns with previous findings in healthy men^22^, indicating their heightened sensitivity to visual sexual cues and a propensity to allocate more attention to such cues. This observation offers a fresh perspective on the potential neurobiological model for HSDD in men. In this proposed model, men demonstrate adequate visual attention to sexual cues; however, these cues might not be effectively relayed to the limbic system, which is responsible for emotional processing. This attentional response to sexual cues therefore seems not to develop into sexual arousal, perhaps suggesting a functional disconnection between sensory/attentional and emotional systems worthy of future study.

This is the first study to directly compare sexual-brain processing in women and men with HSDD and had a number of strengths. All participants were biochemically eugonadal, thereby removing the confounding effect of hypogonadism on our neural and behavioural findings. Regarding the women, all participants were premenopausal with study visits undertaken on days 1–7 of the menstrual cycle (follicular phase) to ensure consistent reproductive hormone levels. This is important as it is well-established that brain activity is altered by fluctuations in reproductive hormones across the menstrual cycle^26^. Moreover, a sample of 64 participants is large for fMRI studies^23^. The participants all interacted with a same sex physician throughout the study, minimising bias associated with investigator sex^24^. The neural response to low-level visual features was adequately controlled for through the use of couple exercise videos. Importantly, this ensured all videos contained one woman and one man with similar levels of movement in both types of videos. The study employed multiple analysis methods, including whole-brain, ROI, and correlations to explore and understand the distinct differences between women and men with HSDD.

A potential limitation of the study is the age difference between the groups, with women on average 9 years younger than the male participants. Although this may have some effect on neural activation, the control > baseline comparison confirmed there was no difference in activation in the ROIs to the control stimuli, suggesting baseline brain activation was similar between the two groups. Moreover, this sample more accurately represents the HSDD population, since younger women tend to be more distressed by their HSDD than older women and so seek help/trials^25^, while HSDD becomes more prevalent as men get older^26^.

The lack of a non-HSDD comparator group limits our ability to extrapolate distinct differences between HSDD and individuals with normal sexual desire (especially in men where there is very limited previous data). Therefore, an important area for future investigation would be an fMRI study comparing sexual-brain processing in women and men with HSDD with individuals with normal sexual desire under the same protocol. Moreover, the present data originated from two previous studies primarily designed to discern variations in brain activity between placebo and hormone administration study visits. Consequently, our power calculation was not initially formulated to detect differences between women and men. Future studies should prioritise incorporating a healthy control group (with normal sexual desire). In addition, all the women were premenopausal which limits the generalisability of our results to post/perimenopausal women. However, this study was the first to directly compare sexual-brain processing in women and men with HSDD under the same protocol, and even with these confounds taken into consideration, the data has shown compelling direct differences between women and men with HSDD. The data provides further evidence and support for the top-down theory of HSDD in women, and the correlational data suggests therapies which may target the hypoactive-limbic system may be effective at increasing positive feelings towards sex in women with HSDD. In addition, the comparatively lower limbic activation in the men is of interest and warrants further study.

Taken together, the observed sexual dimorphism in behavioural and neuroimaging data have significant implications for our understanding of disrupted sexual desire and arousal in women and men. In women, HSDD is likely driven by top-down inhibition of the sexual response. This was shown in the hyperactive inferior frontal gyrus and the correlations implying lower activation in sexual brain regions is associated with lower sexual function on psychometric assessment. Together this suggests therapies targeted at reducing hyperactivity in higher-cortical regions or boosting activation in lower-limbic regions could improve sexual function in women with HSDD. In men, there may be a different model of HSDD, whereby the visual attention to the sexual cues is not effectively relayed to emotional centres involved in the sexual response. Unlike in women, more activation in limbic brain regions in men did not translate to greater sexual functioning on the SADI questionnaire. This suggests that therapies which may target functional connectivity could benefit men with HSDD. Psychedelic therapy has recently been shown to reduce brain modularity^27^, and is thought to enable the formation or restoration of adaptive functional connectivity patterns^28^. This could perhaps be a useful potential intervention for men with HSDD to strengthen the connectivity between the visual and emotional centres of the brain. Identifying this sexual dimorphism has important clinical implications for the development of sex-specific diagnostic and therapeutic approaches for individuals distressed with low sexual desire.

## Methods and materials

To compare neural correlates in distressing low sexual desire in women and men, data was collected from the placebo visits of our parallel studies exploring the effects of hormone administration on sexual-brain processing in premenopausal women and men with HSDD (full methods described in^29,30^).

### Ethics

Ethical approval for this study was obtained from the Riverside Research Ethics Committee (REC ref: 17/LO/1504). The study was conducted in line with the Declaration of Helsinki and International Council for Harmonization Guidelines on Good Clinical Practice. All participants provided informed written consent before inclusion.

### Participants

Adverts were placed across London, in print media, and online inviting premenopausal women and men distressed and/or concerned by low sexual desire for > 6 months to take part. Interested participants were initially telephone-screened and subsequently underwent a detailed medical history and examination screening visit to confirm a diagnosis of acquired and generalised HSDD in accordance with the ICD-11 criteria ^31^. Participants were required to have no current or past psychiatric illness and be free of current medications. Detailed inclusion and exclusion criteria can be found in ^29,30^. Of note, participants were required to be in a stable, communicative, and monogamous relationship for > 6 months. Participants were excluded if they had a history of unresolved sexual trauma, abuse or aggression, use of medications (prescribed or over the counter) or herbal preparations to enhance sexual desire, arousal or performance, or had contraindication to MRI scanning. In addition, participants completed a series of psychometric questionnaires to assist in the diagnosis of HSDD including: ‘Female Sexual Function Index’ (FSFI) and ‘Female Sexual Distress Scale—Desire/Arousal/Orgasm’ (FSDS-DAO) [in women], and ‘Sexual Concerns Inventory-Male’ (SCI-M), ‘Sexual Desire Inventory’ (SDI) and ‘International Index of Erectile Function’ [in men]. The ‘Patient Health Questionnaire-9’ (PHQ-9) and ‘General Anxiety Disorder-7’ (GAD-7) questionnaires were performed to exclude underlying depression and anxiety, respectively. Blood samples were taken to ensure normal health status and exclude any endocrine abnormalities. All participants were biochemically eugonadal, with a normal circulating testosterone level (in men) and premenopausal status (in women) (as reported in ^29,30^).

Following screening and informed consent, 32 premenopausal women and 32 men with HSDD were recruited. All participants completed the protocol and formed the dataset described in the current study.

### Study design

Participants completed questionnaires before and immediately upon completion of the fMRI scan, to assess for any dynamic behavioural effects related to the sexual stimuli and then correlate those changes with the brain activity data (described below). The 'Sexual Desire and Arousal Inventory' (SADI) is a validated multidimensional scale assessing subjective sexual arousal and desire based on 54-descriptors categorized into four domains: evaluative (e.g., sexy, excited), negative (e.g., aversion, resistant), physiological (e.g., tingling, flushed) and motivational (e.g., lustful, alluring)^32^. Participants were provided with the written instruction ‘*please indicate to what extent each word describes how you currently feel*’ and were asked to rate each descriptor on a scale from 0 (‘*does not describe it at all*’) to 5 (‘*describes it perfectly*’). During the imaging session participants underwent a T1 structural scan and an fMRI scan assessing sexual-brain processing.

### fMRI procedure and acquisition

A mirror mounted on the head coil allowed participants to view a screen mounted in the rear of the scanner bore, where visual stimuli were back-projected through a wave guide in the rear wall of the scanner room. Participants wore headphones to receive instructions, and a pulse-oximeter was attached to the participant and connected to a standard data-recording system (AD instruments PowerLab) in the control room. The sexual-brain processing task was a standard validated block design lasting 12 min and consisted of 20-s silent sexual videos alternating with neutral non-sexual exercise videos as a control. Given that women and men require different visual sexual stimuli to become sexually aroused^33,34^, we convened two separate and independent focus groups comprised of 20 healthy heterosexual women and 20 healthy heterosexual men to rate a series of sexual videos taken from commercial adult films. The 10 videos with the highest sexual arousal scores from the female and male focus groups formed the fMRI stimulus sets for the female and male studies, respectively. During the fMRI task, 10 sexually explicit videos (depicting one woman and one man engaging in sexual intercourse) alternated with 10 non-sexual control videos (depicting one woman and one man exercising together). To maintain alertness and task engagement, participants were asked to rate their subjective level of arousal on a 20-point scale using a hand-held button box after each video. The rating period lasted for 5-s and was followed by a 10-s blank grey screen, which provided a baseline/rest condition. As expected, both the women and men rated the sexual videos as more arousing than the control videos (average difference between sexual and control videos: women =  + 8.5 and men =  + 8.8, on a 20-point Likert scale), confirming the effectiveness of the stimulus sets to induce sexual-brain processing.

Imaging data for both groups were acquired using a Siemens Magnetom Trio 3 T scanner with a 32-channel, phased-array head coil. Anatomical images were acquired at the beginning of each scan using a T1-weighted magnetisation prepared rapid gradient echo (MPRAGE) pulse sequence (1 mm isotropic voxels, repetition time [TR] = 2300 ms, echo time [TE] = 2.98 ms, flip angle = 9°, 160 slices, 256 × 256 in-plane FOV, bandwidth = 240 Hz/pixel, GRAPPA = 2. For the acquisition of functional images, a multiband sequence with acceleration factor 2 (similar to the sequences previously validated in ^35^ was used with the following parameters: 3 mm isotropic voxels, TR = 1250 ms, TE = 30 ms, flip angle = 80°, 44 axial slices, bandwidth = 2232 Hz/pixel, GRAPPA acceleration = 2, 192 × 192 mm FOV.

### fMRI data analysis

fMRI data processing was performed using FEAT (fMRI Expert Analysis Tool), part of the Oxford Centre for Functional MRI of the Brain (FMRIB) Software Library (FSL) version 6.0 (www.fmrib.ox.ac.uk/fsl). Registration on to high resolution structural images was carried out using FMRIB Linear Image Registration Tool (FLIRT)^36^. Registration from the high resolution T1 structural image of each participant to standard Montreal Neurological Institute (MNI) 152 space was then further refined using FMRIB's Nonlinear Image Registration Tool (FNIRT)^37,38^. The following pre-statistic processing was applied: motion-corrected FLIRT^36^, non-brain removal using the Brain Extraction Tool (BET)^39^, spatial smoothing (6.0 mm) and 90-s high-pass temporal filtering. All first level models included the extended set of head motion parameters regressors (original parameters, plus derived temporal derivatives and quadratic functions). White matter and cerebral spinal fluid masks were created from each participant's anatomical scans using the FMRIB’s Automated Segmentation Tool (FAST), and the time series from each functional scan was extracted from these masks for use as a regressor of no interest for each participant in each task to further denoise the data. Time-series statistical analysis was carried out using FMRIB’s Improved Linear Model (FILM) with local autocorrelation correction^40^.

The regressors of interest were derived from the onset times of the sexual and control video conditions and were convolved with a gamma function to simulate the hemodynamic response function (HRF). These were used as the main regressors of interest in the general linear model (GLM) with the denoising methods mentioned above as regressors of no interest. The contrasts were defined by each stimulus condition compared to baseline and then also comparing two stimulus conditions of interest, contrasts comparing the sexual and control conditions were our main outcome. A between-subjects model was used to investigate differences in whole brain activation between women and men with HSDD in the sexual > control contrast. Statistical images were set at threshold using clusters determined by Z > 2.3 and a corrected cluster significance threshold at P = 0.05.

### Statistical analysis

Overlap in the similarities of activation patterns were calculated using Dice Coefficients^41^. The thresholded results of the group analyses were binarised and multiplied together to create image masks containing only the voxels that overlap in the female and male groups. Dice coefficients were then calculated using the following equation:

$$(2 * C)/(A + B).$$

where C is the number of voxels in the overlap image, A is the number of voxels in the female group average image and B is the number of voxels in the male group average image. Dice coefficients give an estimate of the spatial replicability of the task across the two different sexes. Dice coefficients are between 0 and 1, where 0 is no overlap and 1 would mean the images are identical.

**Figure 5:**
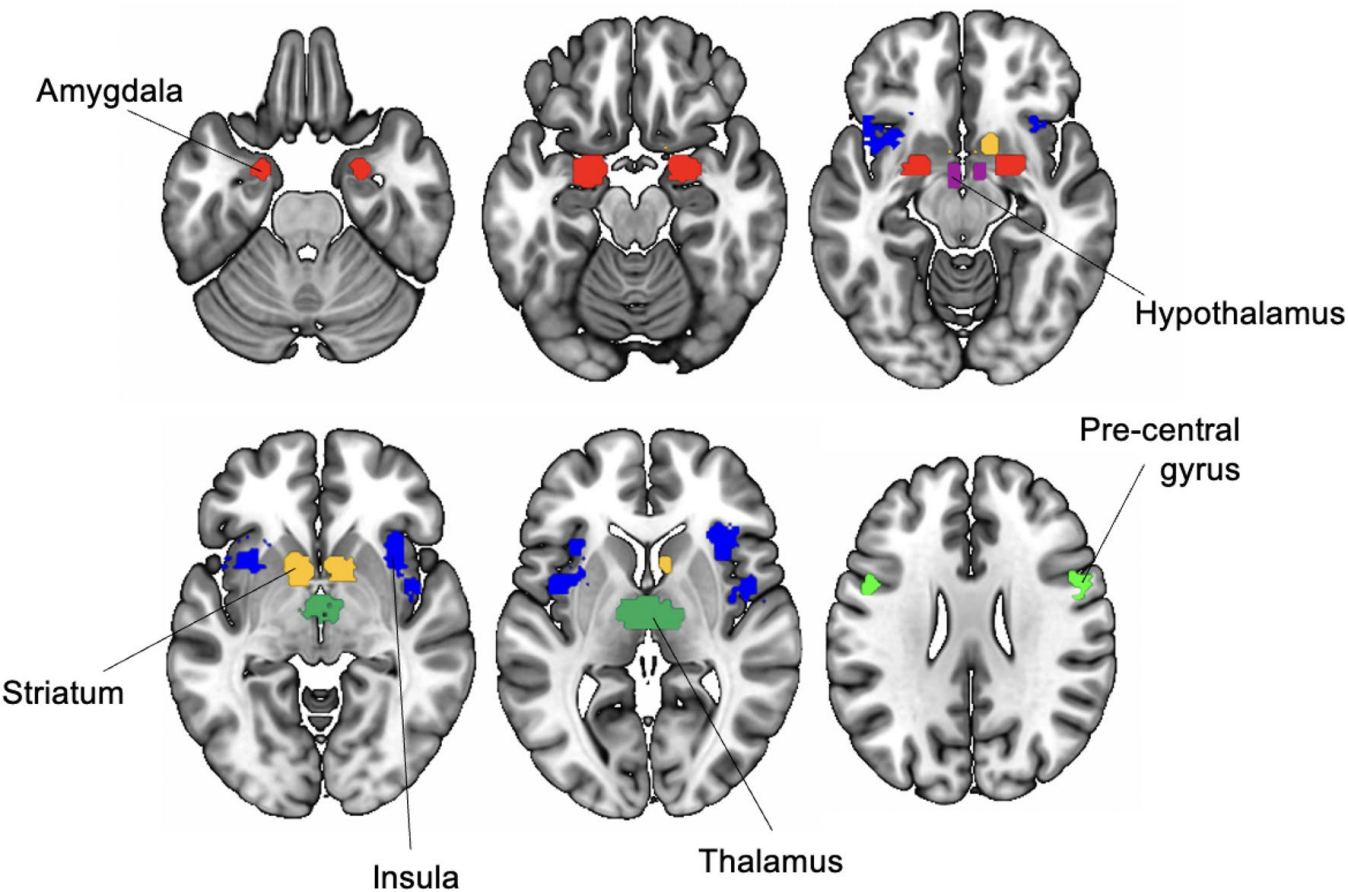
Sex network ROIs. ROIs derived from *Neurosynth* meta-analytic data using the term ‘sexual’.

ROIs employed in the current study were defined using the term ‘sexual’ in the meta-analytic site *Neurosynth* (available at: https://neurosynth.org/analyses/terms/sexual/) for a separate and independent study^42^. MNI152 space masks for the ROIs are available at: https://osf.io/t7g5x/. ROIs are shown in Fig. 5. ROIs were treated as single regions and were not lateralised to left/right.

Data was extracted from the sexual > control contrast within the ROIs and an ANOVA was used to confirm a main effect of sex followed by *post-hoc* unpaired t-tests to compare activation in the ROIs between women and men. To control for general brain activation differences between the sexes, data was extracted from the control > baseline and sexual > baseline contrasts as well. The data from the ROIs (sexual > control) was then correlated using Spearman’s correlation for each of the four SADI domains (using the post-scan ratings). All results were FDR corrected using Benjamini–Hochberg correction ^35,43–46^, treating men and women as separate families.

Power calculations were performed on the original projects based on previous data of BOLD responses in key sexual brain regions. For full power calculations see ^29,30^. The sample sizes used are consistent with previous fMRI research conducting between-subjects analyses^23^.

### Supplementary Information

Supplementary Information.

## Data Availability

Some or all data sets generated during and/or analyzed during the present study are not publicly available but are available from the corresponding authors on the basis of reasonable scientific merit. All data provided are anonymized to respect the privacy of the participants who participated in the study.
