# Supplementary material for: Women and men with distressing low sexual desire exhibit sexually dimorphic brain processing
Source: Sci Rep. 2024 May 14;14:11051. doi: 10.1038/s41598-024-61190-4 (PMC11094107; doi:10.1038/s41598-024-61190-4)
Supplement: Supplementary file 1 — Supplementary Information. [file 41598_2024_61190_MOESM1_ESM.docx]

**Supplementary: Women and Men with Distressing Low Sexual Desire Exhibit Sexually Dimorphic Brain Processing**

Natalie Ertl^#1,2^, Edouard G Mills^#2^, Matthew B Wall^1,2^, Layla Thurston^2^, Lisa Yang^2^, Sofiya Suladze^2^, Tia Hunjan^2^, Maria Phylactou^2^, Bijal Patel^2^, Paul A Bassett^3^, Jonathan Howard^1^, Eugenii A Rabiner^1^, Ali Abbara^2^, David Goldmeier^4^, Alexander N Comninos*^2,5^, Waljit S Dhillo*^2,5^.

^#^Co-first authors

*Co-corresponding/senior authors

^1^Invicro London, Burlington Danes Building, Hammersmith Hospital, London, UK.

^2^Section of Endocrinology and Investigative Medicine, Imperial College London, Hammersmith Hospital, London, UK.

^3^Statsconsultancy Ltd, Amersham, UK.

^4^Jane Wadsworth Sexual Function Clinic, Imperial College Healthcare NHS Trust, London, UK.

^5^Department of Endocrinology, Imperial College Healthcare NHS Trust, London, UK.

**Corresponding authors:**

Professors Alexander N. Comninos & Waljit S. Dhillo

Section of Endocrinology and Investigative Medicine,

Imperial College London,

6^th^ Floor, Commonwealth Building, Hammersmith Hospital Campus,

Du Cane Road, London W12 0NN, United Kingdom.

+44 (0)20 7594 3487 [a.comninos@imperial.ac.uk](about:blank) [w.dhillo@imperial.ac.uk](about:blank)

**
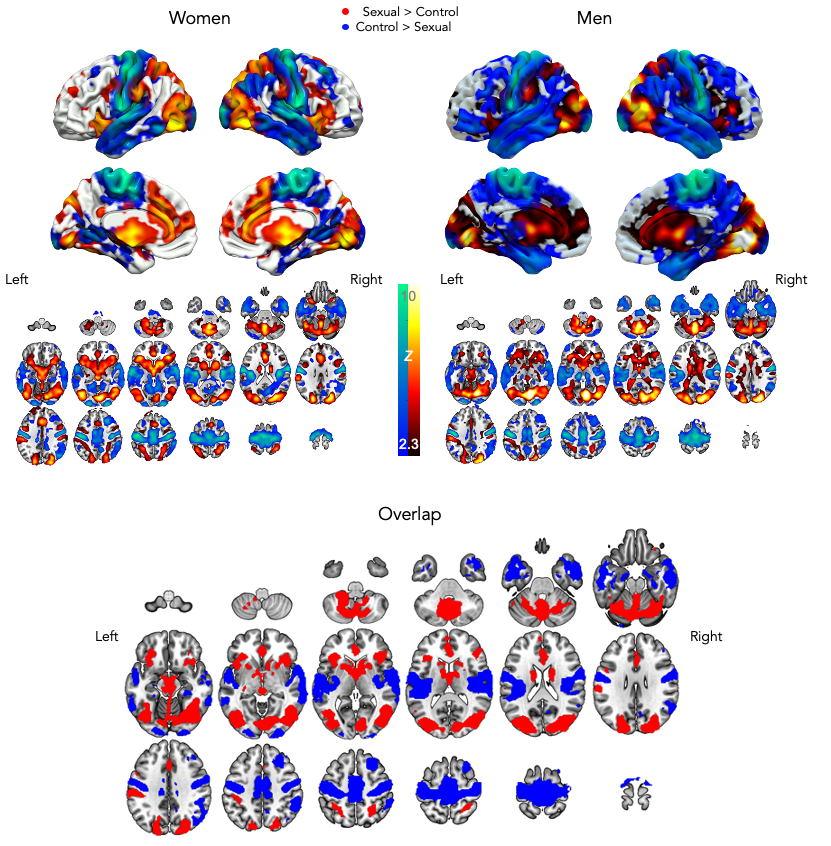
**

**Supplementary Figure 1**. Activation to sexual > control (red/yellow) and deactivation (or control > sexual; blue/green) in the female and male cohort. In the lower panel is the overlap image generated from the binarised masks showing direct areas of overlap in the patterns of activation (red) and deactivation (blue) in women and men with HSDD to sexual > control videos. Results are cluster corrected and thresholded to Z=2.3, P<0.05, N=64 (32 women, 32 men).

**Supplementary Figure 2** A) No difference in activation in regions of the sex network between women (purple) and men (green) was observed when watching control exercise videos, compared to baseline. B) Women had significantly greater activation in the amygdala, hypothalamus, and striatum, than men when watching sexual videos (compared to baseline). P values are FDR corrected for multiple comparisons using Benjamini-Hochberg correction: *P<0.05 *, P<0.01 ** P<0.001***.* Error bars show SEM, N=64 (32 women, 32 men).

|  | **Cluster** | **L/R** | **Voxels** | **Z max** | **X** | **Y** | **Z** | ***p*** |
| --- | --- | --- | --- | --- | --- | --- | --- | --- |
|  | Women, Sexual > Control (Figure 1a) | | | | | | | |
| 2 | Inferior Frontal Gyrus | R | 46479 | 7.05 | 40 | 28 | 4 | <0.0001 |
| 1 | Pre-Central Gyrus | L | 957 | 4.79 | -50 | 4 | 36 | 0.0009 |
|  | Women, Control > Sexual (Figure 1a) | | | | | | | |
| 4 | Insula Cortex | L | 44070 | 8.04 | -38 | -16 | 16 | <0.0001 |
| 3 | Occipital Pole | L | 1225 | 5.67 | -22 | -100 | -4 | 0.0001 |
| 2 | Occipital Pole | R | 996 | 6.19 | 24 | -102 | 8 | 0.0065 |
| 1 | Frontal Pole | R | 605 | 3.41 | 28 | 48 | 4 | 0.0178 |
|  | Men, Sexual > Control (Figure 1b) | | | | | | | |
| 2 | Lingual Gyrus | R | 41013 | 8.78 | 6 | -76 | -2 | <0.0001 |
| 1 | Supramarginal Gyrus | L | 1241 | 5.26 | -56 | -26 | 34 | 0.0001 |
|  | Men, Control > Sexual (Figure 1b) | | | | | | | |
| 5 | Precentral Gyrus | L | 43477 | 7.22 | -46 | -14 | 40 | <0.0001 |
| 4 | Precuneus | L | 1099 | 4.6 | -2 | -60 | 54 | 0.0003 |
| 3 | Frontal Pole | L | 927 | 4.43 | -44 | 44 | -12 | 0.0011 |
| 2 | Occipital Pole | R | 748 | 5.63 | 26 | -100 | -8 | 0.0050 |
| 1 | Occipital Pole | L | 648 | 5.03 | -28 | -96 | -10 | 0.0119 |
|  | Women > Men (Figure 1c) | | | | | | | |
| 4 | Parahippocampus | L | 4850 | 4.55 | -18 | 4 | -18 | <0.0001 |
| 3 | Inferior Frontal Gyrus | L | 1007 | 4.04 | -52 | 20 | 6 | 0.0008 |
| 2 | Superior Frontal Gyrus | R | 671 | 3.88 | 6 | 16 | 62 | 0.0110 |
| 1 | Brainstem | R | 570 | 4.23 | 6 | -28 | -26 | 0.0279 |
|  | Men > Women (Figure 1c) | | | | | | | |
| 2 | Lingual Gyrus | R | 12395 | 6.27 | 12 | -68 | 0 | <0.0001 |
| 1 | White Matter | L | 550 | 3.91 | -24 | 36 | -4 | 0.0330 |

**Supplementary Table 1.** Table of coordinates for group means and direct comparison results from whole-brain analysis. The coordinates are for the maximum Z value within the cluster, these are derived from the cluster list coordinate information from the FSL report and are in MNI-152 space. N=64 (32 women, 32 men).
